# The impact of implementing a psychiatric emergency hotline on the reduction of acute hospitalizations in a Swiss tertiary hospital

**DOI:** 10.1186/s12888-021-03431-8

**Published:** 2021-08-31

**Authors:** Aurélio Restellini, Omar Kherad, Stefan Kaiser

**Affiliations:** 1 Division of Psychiatry, Geneva University hospitals and University of Geneva, Chemin du Petit-Bel-Air 2, 1226 Thônex, Geneva, Switzerland; 2 Department of Internal Medicine, La Tour Hospital and University of Geneva, Geneva, Switzerland

**Keywords:** Adult psychiatry, Triage, Decision making, Choosing wisely

## Abstract

**Background:**

Inpatient treatment is not the most beneficial treatment setting for many patients with psychiatric disorders and overcrowding is a recurrent problem for psychiatric hospitals. Therefore, it is important to develop strategies to limit avoidable inpatient treatment. This study sought to evaluate the impact of an emergency hotline that was developed to better manage psychiatric patients, particularly for identifying those requiring a hospital admission.

**Methods:**

This pre-post intervention quality improvement study compared changes in the management of psychiatric patients’ admission before and after the introduction of an emergency hotline where a specialist in psychiatry examines all inpatient referral from private practitioners.

Main outcomes were the change in proportion of hospital admissions after referral from a private practitioner before and within 3 months after the intervention. Secondary outcomes were the average length of hospital stay, proportion of non-voluntary admission, the time required for triage and the impact of the intervention on treatments’ costs. Fisher’s Exact test was used to test the primary hypothesis of difference in the proportion of hospitalized patients before and after introduction of the emergency hotline. Secondary outcomes were tested with Student’s t-test for continuous variables and Fishers’s Exact test for proportions.

**Results:**

Among 45 admission requests from private practitioners during the 3 months after introduction of the new emergency hotline, 25 (55.6%) were accepted as inpatient treatment, while 20 (44%) were redirected to more appropriate outpatient treatments. There was a highly significant difference from the baseline period during which all 34 requests were accepted (44% vs 100%, *p* < 0.001). In addition, for the patients hospitalized after the introduction of the emergency hotline there was a trend-level reduction of the average length of stay (9.32 days vs 17.35 days).

**Conclusion:**

Implementation of an emergency hotline manage by a specialist in psychiatry for admissions to acute psychiatric wards is feasible and simple to use. Importantly, it allows to significantly decrease the proportion of hospitalizations. Additional studies are needed to assess the generalizability of these exploratory results to other health care settings.

## Background

Hospitalization in psychiatry is not always beneficial and can even be harmful in the medium/long term [1–6]. Furthermore, psychiatric patients are usually admitted to hospital on a voluntary basis, but in some situations, they may be committed as an involuntary patient [7]. In addition, hospital treatment and management of psychiatric patients is one of the most expensive care that can be provide to psychiatric patients [8, 9]. Despite highly developed outpatient networks in many developed countries, overcrowding of inpatient psychiatry services remains a common problem [10–13]. This overcrowding is problematic not only in terms of inadequate conditions with respect to rooms and other facilities, but also in terms of the quality of care. It is now well established that overcrowded units have negative impact on staff who are less able to offer optimal care to patients [14–16].

Considering a significant increase in psychiatric disorders over the last two decades [17–20], it is important to assess different strategies to limit avoidable inpatient treatment, in particular the use of triage instruments [21, 22]. Avoidable inpatient treatment refers to situations in which outpatient treatment can be provided to the patient without at least equivalent outcomes. Outpatient treatment refers to a range of services, including mobile crisis intervention teams and acute day clinics for which non-inferiority has been demonstrated [23, 24]. In addition, for some conditions such as borderline personality disorder it is specifically recommended to avoid inpatient treatment whenever possible [25]. The reduction of unnecessary interventions is a broader objective in medicine, which is advocated by the Choosing wisely campaign [26]. Several models exist and have proven their effectiveness for this purpose [27, 28]. By focusing on day-hospitals or intensive treatment in the community, these models reduce the number of hospital admissions [7, 27]. An important question is how to address acutely ill patients to the appropriate care setting, highlighting the need of an appropriate triage. The identification of those patients needing hospital admission as the most intensive intervention is a critical task for triage that needs the development of appropriate instruments.

To date, despite compelling literature on triage instruments in different emergency medical specialties [29–31]. Triage instruments in psychiatry have mainly been developed for assessing patients arriving at psychiatric emergency departments with a focus on the detection and management of acute life-threatening conditions [32, 33]. A Swiss research group has implemented a more comprehensive triage system to reduce the number of hospitalizations and has recently published positive results [34]. However, in addition to a triage by telephone these authors emphasized face-to-face evaluations and their approach may thus be relatively resource-intensive.

**Fig. 1:**
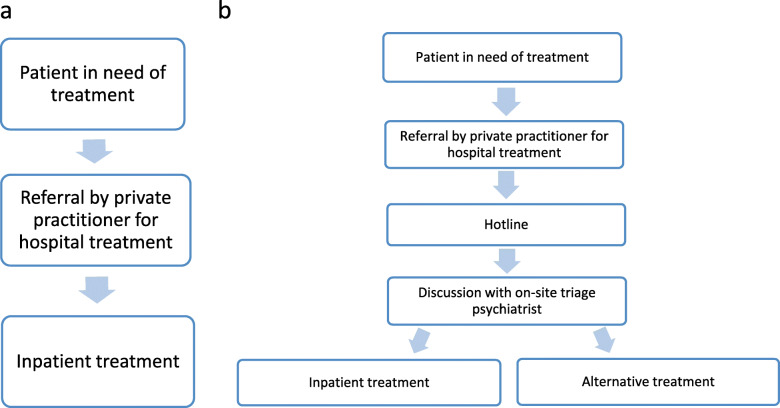
a: Admission process before March 2019*.* b: Admission process since March 2019

Before the start of the present study, there was no triage instrument in Geneva, Switzerland, and the admission to the psychiatric hospital was left to the discretion of the treating outpatient physician (Fig. 1a). The emergency hotline allowed the treating physician working in private practice, wishing to hospitalize a patient, to discuss the clinical situation with a specialist psychiatrist able to propose alternative solutions whenever it was possible in order to limit inappropriate hospital admissions.

The present study assessed the impact of the implementation of an emergency hotline in psychiatry. The aim was to evaluate the benefit of emergency hotline on the rate of hospitalizations in acute psychiatric wards and the number of days spent in hospital (days per patients and overall days). The hypothesis underlying this second objective is that with better indications for in-hospital treatment, treatment goals could already define before admission and the hospitalization could be limited to attaining these goals.

## Methods

### Design

We performed a pre-post intervention quality improvement retrospective study to compare changes in the rate of psychiatric patients’ admission (number of hospitalizations/number of referrals for hospitalization from private practice) in acute psychiatric ward before and after the introduction of an emergency hotline and thus measure the impact of the intervention. The baseline period included referrals for hospitalization that occurred from March to May 2018 and the post intervention period also analyzed referrals that occurred over the same 3-months period in 2019. The selection of the same period of the year (March to May) permits to avoid any bias induced by seasonal fluctuations. We decided to analyze only two years (2018–2019), because our hospital underwent important organizational changes during the year 2017 and therefore data from the period March to May prior to 2018 are not directly comparable. We conducted an additional analysis by using another baseline time period, measuring the admission rate during another 3-month period just before the intervention (Dec 2018 to Feb 2019).

### Setting and study population

The Swiss health care system is based on a basic compulsory health insurance for anyone residing in Switzerland with state support for those who cannot afford to pay themselves. This insurance covers multiple medical treatments, including psychiatric care, whether it is an outpatient clinic, a day hospital or an inpatient treatment. With 578 adults psychiatrists and child psychiatrists registered in 2019 [35], Geneva has one of the highest density of psychiatrists per capita in the world [36]. In addition to the numerous private practices in the canton, several private and public outpatient care centers are available. The Geneva University Hospitals (Hôpitaux Universitaires de Genève - HUG) alone have three specialized centers for adult psychiatry providing care and follow-up for more than 2500 patients. A mobile team is also available for adult psychiatry. This program, based on the Assertive Community Treatment (ACT) model of care [37], is intended for people with severe and persistent mental disorders, who are difficult to reach or to maintain in a traditional treatment program and who show a marked deterioration in their social functioning and daily living skills [38]. During the study period the adult psychiatry division had three acute inpatient wards of 14 to 16 beds to which all patients were referred for hospitalization. The staff consists of 3 to 4 nurses per shift with the option to add additional resources for exceptional cases, 2 to 3 psychiatrists in training and 1 specialist in psychiatry. These units often operated at occupancy rates exceeding their maximum capacity, which was of the main motivations for the implementation of the intervention studied here.

All patients referred to an adult acute psychiatric ward at HUG following a request from a private practitioner (general practitioner, psychiatrist or psychologist working in private practice) during the studied periods and during opening hours (8 a.m. to 6 p.m. on working days) were included. There were no other inclusion criteria. Patients hospitalized directly from the emergency ward and patients sent by psychiatric department’s outpatient units were excluded because these patients did not have to go through the emergency hotline procedure. No other exclusion criteria were applied, concerning for example diagnosis, reasons for hospitalization or socio-demographic characteristics.

### Intervention

An emergency hotline to control hospitalization was introduced in March 2019. The designation of an emergency hotline, available from 8 a.m. to 6 p.m. on working days (5/7), allowed the treating physician or psychologist working in private practice, wishing to hospitalize a patient, to discuss the clinical situation with a specialist psychiatrist on site.

This psychiatrist on site was able to propose alternative solutions (intensive outpatient follow-up for example) in order to limit inappropriate hospital admission. It is important to note that for adult psychiatry inpatient units, outpatient centers and the ACT team all belong to the same division ensuring that inpatient psychiatrists will have excellent knowledge of alternatives to hospitalization. The on-site team responsible for the hotline was composed of 6 specialists in psychiatry. All of these specialists worked full-time and were In charge of one inpatient ward. They were responsible for the hotline for 5 consecutive days in addition to their other clinical duties. No additional staff was hired to cover the hotline.

The procedure that has been put in place to facilitate hospitalization consists of a three-step assessment (Fig. 1b):

1. A patient requiring inpatient psychiatric treatment must be assessed by a specialist in psychiatry. Thus, any patient who has not previously seen a psychiatrist must be referred to one, whether it is the private psychiatrist in charge of the patient’s care or the psychiatric emergency department available 24/7. If a doctor, or caregiver, who is not a specialist psychiatrist refers a patient to the psychiatric hospital, then he will be redirected to the psychiatric emergency department for an initial evaluation by a specialist.
2. Once this patient is assessed by a specialist, then the specialist will contact the psychiatrist on site at the psychiatric hospital. The situation will then be discussed over the phone with or without the support of the patient’s digital file to determine the best course of action. This means that the request for hospitalization is discussed by two psychiatric specialists; the one who refers the patient and the one who receives the patient (on-site psychiatrist). The rationale behind this double assessment is as follows: Although both specialists are able to assess the severity of the symptoms, the private psychiatrist do not necessarily have knowledge of the full range of services available for his patient (intensive ambulatory care for example).
3. This psychiatrist on site can propose alternative solutions whenever it is possible to limit inappropriate hospital admission.

However, this intervention is not used to manage requests for hospitalization made by outpatient units belonging to the adult psychiatry division. Nor it is used to process transfers of patients between inpatient units belonging to the adult psychiatry division.

In case of any uncertainty or a particularly complex situation, the patient can be physically assessed on site by the psychiatrist and subsequently redirected to another treatment if necessary. It is only after these steps of evaluation that the patient can be admitted to the hospital. Considering the heterogeneity of symptoms that patients may show, no criteria limiting hospitalizations based on symptoms alone were established. Thus, although taken into consideration in the evaluation made by the attending physician, no patient was discriminated based on his or her diagnosis. Additional factors were considered, including the available family support network, the current follow up, etc.

For the implementation of this hotline, the procedure regulating admissions to the adult psychiatry division was adapted and made available to all staff via the division’s intranet. The psychiatrists on site did not receive any specific training, but a senior psychiatrist was continuously available to discuss any questions. A communication was made by email to the different actors of the healthcare network in Geneva, explaining the new admission procedure to prevent any misunderstanding.

### Outcome and data collection

The primary outcome was rate of psychiatric patients’ admission (number of hospitalizations/number of referrals for hospitalization from private practice) before March 2019 and after March 2019.

Secondary outcomes included the average length of stay in hospital before and after the intervention, the rate of voluntary vs involuntary admissions before and after the intervention, the time required for triage, categories of reoriented inpatient treatment, the impact of diagnostic on admission, the impact of the intervention on treatments’ costs.

Data regarding the number of hospital stays following a referral from private practice and data concerning hospitalizations in adult acute psychiatric care as well as the average time spent in hospital (days per patient and total number of days) and final diagnosis were systematically collected from the electronic health records from HUG.

More specific data during the intervention period was collected directly through the new the emergency hotline: the origin of the admission request, the clinical motivation for hospitalization, the admission status, the referring practitioner, whether or not a second evaluation on-site was needed, the time required for the admission management and the final orientation.

### Statistical analysis

Analyses were performed to compare the admission rate following a private practice request before and after the introduction of the emergency hotline. Fisher Exact test was used to test the primary hypothesis of difference in the proportion of hospitalized patients before and after introduction of the emergency hotline. Differences in mean length of stay per patient was then compared using a two sample Student’s t-test. Differences in the rate of voluntary vs involuntary admissions before and after the intervention and the impact of diagnostic on admission were also compared using a Fisher Exact test. Statistical analyses were performed using R version 3.6.2 (R Foundation for Statistical Computing, Vienna, Austria). *P* values < 0.05 were considered statistically significant. We estimated the required sample size based on Fisher exact test for our primary hypothesis and assuming an admission rate of 100% following a hospitalization request in 2018 (based on previous yearly audit results performed at HUG). The power analysis showed that 34 patients in each group would be needed to reach 80% power to detect a drop of 20% in admission rate after the intervention, with a significance level of 0.05. A two-sided test was chosen in order to be able to detect a non-expected increase in admission rates.

*Ethics Considerations.*

This study was based on a retrospective use of clinical data authorized under the art. 34 LRH (lack of consent) by the Geneva Cantonal Commission for Ethics in Human Research (CCER) on November 2, 2020 (Project-ID 2020–02103). We confirm that all methods were carried out in accordance with relevant guidelines and regulations.

## Results

### Admissions

During the two 3-months periods of interest in 2018 and 2019, 566 patients were overall hospitalized in psychiatry division at HUG (309 in 2018 and 257 in 2019).

**Fig. 2:**
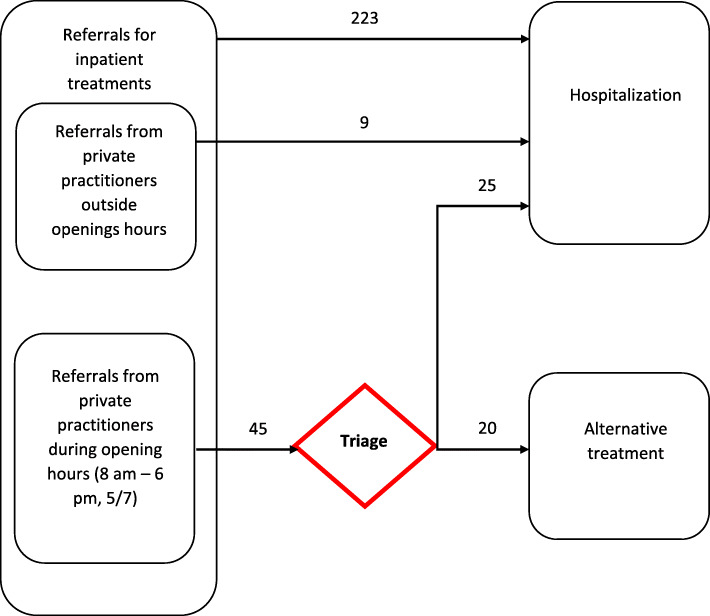
Emergency hotline’s impact on hospitalizations

Concerning our primary outcome, *N* = 88 (*N* = 34 in 2018 and *N* = 54 in 2019) hospitalizations were requested by private practitioners after exclusion of patients according to our selection criteria. Regarding the 54 requests by private practitioners in 2019, only 9 of them were addressed outside the emergency hotline’s openings hours and were thus directly admitted. Therefore, only 45 requests from private practitioners were handled by a psychiatrist on site, through the hotline and included in the final analysis (Fig. 2).

After the implementation of the emergency hotline, only 25 patients were admitted out of 45 hospitalization requests (55.6%) compared to 34 (100%) during the baseline period (Table 1). This difference in hospitalization rates was highly significant (*p* = < 0.0001).

**Table 1:** Primary and secondary outcomes

	Without emergency hotline assessment (2018)	With emergency hotline assessment (2019)	Test statistic and *p*-values
Referrals by a private practitioner	34 Referrals	45 Referrals	N/A
Hospitalizations after a referral by a private practitioners	34 (100%) Hospitalizations	25 (55.6%) Hospitalizations	Fisher Exact test: *p* = < 0.0001
Days spent in hospital in total	590 days	233 days	N/A
Average length of stay per patient	17.4 days (SE = 3.88; SD = 22.6)	9.3 days (SE = 1.34; SD = 6.7)	t = 1.7152; df = 57 *p* = 0.0917
Involuntary patients referred vs admitted	9 patients vs 9 patients	7 patients vs 3 patients	Fisher Exact test: *p* = 0.062

*Note: N/A = Not applicable, as no statistical test was used*

In an additional analysis, we addressed the time period directly preceding the post-intervention period (December 2018 – February 2019). During this time period 35 patients were referred private practitioners and all of these patients were hospitalized. There was a significant reduction from this time period to the post-intervention time period (p = < 0.0001).

### Days spent in hospital

590 days of hospitalization in acute psychiatry units for patients referred by private practitioners were recorded during the baseline period compared to 233 days after the intervention (Table 1). The average length of stay was numerically lower after the intervention (9.32 days, SE = 1.34, SD = 6.74) compared to baseline period (17.35 days, SE = 3.88, SD = 22.64), *p* = 0.0917, without reaching statistical significance.

### Voluntary vs involuntary admissions

The majority of the situations handled by the emergency hotline were voluntary hospitalization requests. During the baseline period 9 referrals from private practitioners were for involuntary hospitalization, all of which were accepted (Table 1). There were 7 involuntary admission requests after the intervention, 4 did not require hospitalization after the triage and were addressed to intensive outpatient treatment. This reduction in the rate of involuntary admissions after referral from a private practitioner did not reach a significant level (Fisher’s exact test, *p* = 0.062).

### Time required for triage

During the intervention, time spent managing the requests took less than 5 min in 2 cases, between 5 and 15 min in 16 cases, between 15 and 30 min in 17 cases, and 30 and 60 min in 6 cases. Only 4 cases took more than an hour to be handled. Overall, about 78% of the situations could be managed in less than 30 min. Among the 45 hospitalizations requests processed by the emergency hotline, only one required a face to face evaluation to decide whether or not a hospitalization was necessary. After this evaluation, the patient was referred to more adequate follow-up and was not hospitalized.

### Alternatives to hospitalization

Out of the 20 cases over 45 requests which did not result in hospitalization during the intervention period, we were able to identify two categories of requests (Fig. 2):

- 13 of them were oriented to an appropriate intensive outpatient treatment, mainly the crisis teams of the department’s outpatient units.
- 7 of them had not been seen by a psychiatrist (patient referred by a psychologist or a general practitioner requesting hospitalization). After an evaluation by the attending physician, these hospitalizations could be avoided through a more adequate outpatient follow-up.

### Clinical motivations for hospitalization

Multiple clinical motivations for hospitalization were reported, ranging from suicidal thoughts, anxiety disorder, mood disorder, substance abuse, agitation, delusional ideas, social issues and personality disorders (Table 2). None of these complaints stood out from the others in terms of frequency, precluding any comparison between specific symptoms and reorientation of inpatient treatment request.

**Table 2:** Clinical motivations for hospitalization

Clinical motivations for hospitalization – 2019	Admitted	Not Admitted
Suicidal thoughts	2	4
Anxiety	0	2
Personality Disorder	3	1
Social issues	2	2
Substance abuse	1	2
Agitation	1	3
Delusional ideas	7	1
Mood disorder	9	5

### Impact on treatments costs

The redirection of the 20 hospitalization requests resulted in a reduction of at least CHF 111,840 in health care costs induced by inappropriate hospitalization. This estimation is made by calculating the cost difference between inpatient and intensive outpatient care (1000 CHF per day vs 400 CHF per day) while assuming a likely length of hospitalization corresponding to the average length of stay per patient after our intervention (600 CHF × 20 × 9.32). On the other hand, the implementation of the hotline required time spent by the doctors on site. This tool is therefore not without cost either: our data show that the time needed to handle calls from private doctors in 2019 (45) was approximately 23 total hours over the whole time period. These 23 h were included in the normal working time of the specialists in psychiatry and are therefore an estimate of the costs incurred with the specialist not being available for other duties during that time. In order to avoid underestimating the costs of the hotline, we used the upper end of each time category in our data. After that, we used the data provided by the canton of Geneva regarding the salary of a medical specialist in the hospital per hours, to which we added social charges (23%) and indirect costs (30%). Thus, we can estimate the cost of the hotline which corresponds to a total of 2428 CHF over the three months study period.

## Discussion

To our knowledge this study is the first to evaluate the impact of an emergency hotline for admission to acute psychiatric wards using an emergency hotline staffed with a specialist psychiatrist. Our results suggest that implementation of this simple emergency hotline is feasible and can reduce the number of hospitalizations following a referral from a private practitioner, with a highly significant decrease of in hospital admissions. We also observed a trend-level decrease in the average length of stay for patients hospitalized after referral. The time required to manage any situation proved to be short and almost 80% of situations could be managed in less than 30 min. As mentioned above, a Swiss research group has already implemented and tested a triage instrument in the form of a specific unit designed for face-to-face assessment [34]. Other triage tools have been used in psychiatric emergency services [21, 33]. They allow the evaluation of the urgency of the situation taking into account the severity of the disorder, the risk of violence, the socio-demographic factors, etc. Although essential, these models are not completely similar to our intervention. The differences between these triages instruments and the emergency hotline implemented in terms of means deployed and costs incurred are significant, in particular with respect to the limited resources required for the emergency hotline presented here.

Given the clear reduction in hospitalization of referred patients, it is an important question on which basis the psychiatrist on-site was able to make the decision not to hospitalize the patient. One potential decision parameter is the clinical motivation for hospitalization or diagnosis, because inpatient treatment is recommended only in exceptional cases for certain disorders, for example borderline personality disorder. However, our data show no significant effect of diagnosis on admission rate. We did not have quantitative data on illness severity, which could be relevant criterion. Another explanation for the lack of effect of diagnosis may be related to the fact that training of inpatient psychiatrists and the referring private practitioners is very similar, thus resulting in a similar evaluation of the clinical aspects of the case. However, these results are in contrast with the international literature which has shown that the severity of a psychiatric illness is positively correlated with an increase in hospitalizations rates and the length of stay in hospital [39, 40]. Other factors also influence the increase in hospitalizations rates, such as socio-demographic characteristics of individuals (e.g., living alone and being unemployed) [41]. The influence of the socio-demographic background of the subjects observed in our study could not be measured due to the lack of available data on this item. Nevertheless, in almost half of the cases, the indication for hospitalization was eventually considered as not adequate despite comparable training of referring and triaging psychiatrist. This observation raises the question of other unmeasured factors influencing the appropriate referral to hospitalization. One critical issue may be the knowledge of the available psychiatric facilities, in particular for intensive outpatient and assertive community treatment. These programs have been strongly developed in Geneva over the year preceding the present study. Therefore, psychiatrists that have worked in private practice for some time may have lacked knowledge in term of psychiatric facilities in their city. In contrast, the psychiatrist on-site had excellent knowledge, because all the facilities belong to the same division.

Thus, the emergency hotline became a tool for coordinating the patient trajectory or simply advice from experts on the currently existing mental healthcare system. Therefore, this study brings another perspective on the management of the psychiatric care system and hospital admissions. Indeed, with this lack of knowledge of the healthcare system in place and the need to discuss situations that are complex and difficult to manage alone, it seems essential to improve communication between private practitioners and hospital doctors. Here, we only focus on the reduction of admission rates, but the present results suggest that this form of hotline for coordinating patient trajectories could have a broader use including the optimal orientation of patients without referral for hospitalization. We have not received any complaints about the implementation of such a procedure. On the contrary, the possibility to discuss complex clinical situations was well received by the doctors working in private practice.

However, it is important to note that addressing these situations has also been made easier by the mental health services existing in Geneva. Implementing the same hotline in a place where outpatient mental health services are non-existent would probably not have the same effect on the reduction of hospitalizations observed in our study. Thus, this aspect must be taken into account in the generalization of this finding.

As mentioned above, the emergency hotline that has been implemented was not without cost. However, the average time for managing these triage situations proved to be short and we estimated the total cost of the hotline to be approximately 2428 CHF over the three months post-intervention period. Nevertheless, a decrease in healthcare costs was also estimated at 111′840 CHF following our intervention, showing a significant impact on the reduction of healthcare costs. Additionally, the hotline proved to be remarkably simple to use because only one doctor had to be involved in the management of admissions. It was also fairly easy to find people who could take on the emergency hotline responsibility, since the task was shared among psychiatrist residents from the hospital. Thus, no training was necessary, and no new positions had to be created, as these doctors were experts in their field and were perfectly familiar with the healthcare system in place in Geneva. Moreover, in view of the impact of our emergency hotline on the number of hospitalizations, we are certain there is need for a psychiatric triage system. Although this study focused on the clinical aspect and the decisions made when hospitalization requests are processed, we believe it is important to reflect on the impact of our emergency hotline when it is put into an economic and public health perspective.

With hospitalization costs reaching approximately 1000 CHF per day, versus about 400 CHF per day for intensive outpatient clinical care, inappropriate hospitalizations can have considerable effects on health care costs. Reducing treatments and medical interventions are of utmost importance today if we want to maintain and improve the quality of care available. At a time when healthcare costs are constantly rising [42], and in a context of an increasingly unstable global economic situation, it seems imperative that these issues be taken into consideration and that we introduce such instruments into the management of psychiatric hospitalization. Reducing costs by limiting inappropriate hospital admission has become a priority and is one of the preferred themes of the *Choosing Wisely* campaign across the world that seeks to help physicians and patients engaging in conversations about unnecessary procedures [26]. Sharing evidence around overuse of hospitalization in psychiatry is essential in raising physician awareness and encouraging behavior change.

### Limitations of study

This study has several limitations. The first limitation is the very small sample studied and the short observation period precluding firm conclusions. It is seems reasonable to say that a longer time period is needed to fully analyze a process as complex as the flow of hospitalizations in psychiatry. Unfortunately, an observation over a longer period of time could not be reproduced due to the several factors significantly modifying the functioning of the existing healthcare system (in particular the emergence of SARS-Cov-2 in early 2020). The absence of significant differences in most secondary outcomes can be explained by a lack of power and subgroup analyses were impossible because of the sample’s size. We think that this kind of emergency hotline is probably more effective on specific psychiatric disorder, as borderline personality for example, and warrants furthers analyses.

Another limitation is that due to the anonymization of the data at the time of their collection, we were unable to follow the evolution of the patients who benefited from our intervention. We were therefore unable to show whether these patients showed an improvement in their condition following a more appropriate referral, or the opposite, a worsening of their psychiatric symptoms. For the same reason, we could not analyze possible sociodemographic and clinical differences between the patients referred during the different time periods, because no specific data were collected at the time of collection. Therefore, the differences between time periods observed in the study could also be affected by patient characteristics. However, there were consistent differences between the post-intervention period and the same time period in 2018 as well as the directly preceding months. Therefore, we probably cannot explain the observed effects with an abrupt change in characteristics of referred patients when the emergency hotline was introduced.

Finally, we have not established a fully standardized method for the triage of hospitalization requests. Thus, the outcome of the requests for hospitalization was dependent on the attending person in charge of the call. Therefore, this system, which can require time and investment on the part of the psychiatrist on site, is dependent on the specialist’s proper performance. It might be appropriate for future research to define precise algorithms, allowing the subjective influence of the sorting doctor to be reduced.

## Conclusion

The use of an emergency hotline psychiatric ward is feasible, simple and seems effective to reduce the proportion of hospitalization. The investment to implement the triage tool seems small compared to the benefit on the management of admissions in acute care psychiatric units. Moreover, beyond the practical aspect and the effect on the number of hospitalizations, this intervention has undoubtedly a high impact on health care costs, making it an essential tool in the management of health care systems when viewed from a public health perspective. Future research should aim to further elucidate the relationship between this triage tool and psychiatric patient management in this specific setting using consistent methodological approaches.

## Data Availability

The data used and analyzed during the current study are available from the corresponding author on reasonable request.

## Abbreviations

HUG: Hôpitaux Universitaire de Genève
CAPPI: Centre Ambulatoire de Psychiatrie et Psychothérapie Intégrée
ACT: Assertive Community Treatment
CCER: Cantonal Commission for Ethics in Human Research
